# Rewiring Attention: Virtual Reality and Brain–Computer Interfaces in the Rehabilitation of Unilateral Spatial Neglect

**DOI:** 10.3390/jcm15031036

**Published:** 2026-01-28

**Authors:** Alix Gouret, Alexandre Delaux, Solène Le Bars, Sylvie Chokron

**Affiliations:** 1 Integrative Neuroscience and Cognition Center, Centre National de la Recherche Scientifique, Université Paris Cité, 75006 Paris, France; 2 Research and Innovation Department, Capgemini Engineering, 92130 Issy-Les-Moulineaux, France; 3 Institut de Neuropsychologie, Neurovision et Neurocognition Hôpital Fondation Adolphe de Rothschild, 75019 Paris, France

**Keywords:** unilateral spatial neglect, brain–computer interface, rehabilitation, neurotechnology, virtual reality

## Abstract

Unilateral spatial neglect (USN) is a complex cognitive syndrome frequently observed after stroke. Characterized by a failure to attend, respond and orient to stimuli on the side opposite the brain lesion, USN significantly impairs patients’ functional independence and presents significant challenges for rehabilitation. Current rehabilitation strategies often fall short in addressing the heterogenous manifestations of USN across perceptual modalities due to limited ecological validity, patient engagement and adaptability to individual needs. Recent advances in neurotechnologies such as virtual reality (VR) and brain–computer interfaces (BCIs) offer promising avenues for overcoming these limitations. These tools enable top-down rehabilitation strategies that directly engage cognitive recovery mechanisms to promote neuroplasticity, and support adaptive interventions tailored to individual profiles. This narrative review explores recent developments and future prospects of VR and BCI technologies in the rehabilitation of USN, both individually and in combination. After outlining key features of USN to frame rehabilitation challenges, it examines VR, BCI, and their integrated applications in this context. While there is growing evidence supporting VR interventions efficacy in enhancing conventional strategies and alleviating USN symptoms, research on BCI applications in this context is still emerging. Nevertheless, insights from broader neurorehabilitation research suggest that combining VR and BCI holds significant promise for advancing cognitive rehabilitation and addressing USN-specific challenges. To illustrate the transformative value of advanced USN interventions, we present a concrete example of a VR-BCI integrated rehabilitation framework in the making, designed to provide a comprehensive and personalized therapeutic approach, bridging technological potential with clinical rehabilitation needs.

## 1. Introduction

Stroke is a leading cause of adult-acquired disability worldwide, affecting over 100 million people [1,2]. Its neurological consequences are highly variable, ranging from motor deficits to complex sensory or cognitive impairments, depending on the location and extent of the brain lesion [3]. This anatomical diversity complicates the development of standardized rehabilitation protocols, highlighting the need for flexible, individualized therapeutic approaches [4]. Among these disabling consequences, unilateral spatial neglect (USN) is one of the most frequent and challenging cognitive syndromes, affecting approximately 30% of stroke survivors [5]. Characterized by a failure to attend or orient to stimuli on the side opposite the brain lesion [6], USN severely affects daily activities and often impedes overall post-stroke rehabilitation outcomes [7,8]. This deficit is not due to primary motor or sensory impairments. Rather, it reflects a disruption of higher-order attentional control mechanisms, commonly interpreted through models distinguishing goal-directed (top-down) and stimulus-driven (bottom-up) processes [9]. These are mediated by distinct neural networks, whose dysfunction contributes to the persistence and variability of USN symptoms [9]. Conventional interventions for USN, while beneficial, can be limited in their ability to provide the intensive, engaging, and ecologically valid training required for meaningful and sustained recovery of these processes [10]. The pervasive nature of USN impairment across perceptual modalities and behaviors underscores the need for comprehensive rehabilitation strategies aimed at maximizing functional recovery across a diverse population of patients. In that regard, recent stroke rehabilitation literature indicates a paradigm shift [11], promoting top-down therapeutic strategies to place the brain at the very center of the recovery process [12]. Historically focused on bottom-up approaches primarily targeting motor recovery [13], modern neurorehabilitation research now explores holistic approaches that recognize the intricate interconnections between motor and cognitive functions and actively engage cognitive recovery mechanisms [14,15].

To that end, brain–computer interfaces (BCIs) have emerged as a particularly suitable tool, promoting neuroplasticity and functional recovery through the integration of brain signals real-time analysis in the therapeutic process. They are particularly valuable in stroke rehabilitation for patients with severe motor and cognitive impairments, overcoming limitations of conventional therapies [13]. By opening a new and immediate channel for interaction with the outside world through neural activity, BCIs offer a unique pathway to re-engage disrupted brain networks and support recovery even in cases of minimal physical responsiveness.

Alongside the reactivation of neuroplastic recovery mechanisms, BCI technology also intrinsically paves the way for personalization of the therapy by tuning the rehabilitation process to individual biometrics [16,17]. This is one of the key aspects of modern medicine holding considerable promise to enhance conventional therapy [18]. To fulfill that purpose, medical research is also harnessing another promising technology: virtual reality (VR). VR enables embedding any standard rehabilitation protocols within fully controlled and personalized virtual environments that can be precisely tailored to individual patient needs [19]. Additionally, its immersive nature, combined with the ability to minimize distractions and gamify therapeutic exercises, shows great potential in facilitating skill transfer and improving patient engagement [20,21].

Interestingly, recent developments in BCI and VR technologies have made considerable progress toward user-friendly and versatile designs, such that these neuro-technologies are now commonly deployed beyond the research laboratory context. Facilitated by decreasing equipment costs and increasingly powerful computational tools, they have steadily made their way into the medical device sector. These advances align with a broader vision for clinical rehabilitation: making therapy more accessible, fostering patient autonomy, and enabling the use of integrated all-in-one tools that can extend therapeutic interventions beyond traditional clinical settings (e.g., home-based interventions) [22,23,24,25]. In particular, BCIs based on electroencephalography (EEG) have gained traction due to their portability and non-invasiveness, making them especially suitable for a wide range of therapeutic applications [26]. Recent studies have shown that integrating VR with EEG-based BCIs can significantly enhance neuroplasticity and lead to improved functional outcomes in stroke rehabilitation [27].

Although recent technological advances have promoted top-down approaches in stroke neurorehabilitation, research targeting cognitive impairments (particularly in complex conditions such as USN) remains underrepresented. This gap is critical given the high prevalence and clinical implications of cognitive deficits following stroke [28,29]. The USN case exemplifies how cognitive impairments, traditionally addressed through low-tech interventions that often lack personalization and ecological validity, could benefit from more adaptive technology-enabled rehabilitation strategies that align with the current shifts in therapeutic approaches.

In this narrative review, we aim to bridge recent development on VR and BCI technologies for USN with emerging trends in neurorehabilitation and pave the way for integrated solutions that would enhance cognitive recovery. After outlining key features of USN to frame rehabilitation challenges, we provide an overview of VR and BCI approaches and examine combination endeavors. We discuss existing gaps and propose research directions to support ecologically valid and patient-centered interventions, as well as their clinical translation.

## 2. Unilateral Spatial Neglect

### 2.1. Definition and Characteristics

#### 2.1.1. Clinical Scope

Unilateral spatial neglect (USN), also known as hemineglect, spatial neglect or hemispatial inattention, is a complex neuropsychological syndrome. This condition is characterized by a failure to respond to, report, or orient toward stimuli presented on the opposite side of a brain lesion, typically following a stroke [6]. USN manifests in part as a selective attentional bias. Patients typically show hyper-attention to stimuli in the ipsilesional hemispace, along with a deficit in both reorienting and orienting attention to the contralesional hemispace [30]. Importantly, this deficit is not attributable to primary sensory or motor impairment and rather reflects a disruption in higher-order attentional processes [6]. As these processes rely on distributed and functionally diverse neural networks within the central nervous system (detailed in the next section), such disruption may account for the complex nature of USN.

Consequently, USN manifests through a heterogeneous range of clinical symptoms across patients [31]. First, USN affects a wide spectrum of behaviors, often causing motor, sensory, representational disorders, singularly or in combination. Motor neglect is characterized by reduced spontaneous movement or action toward the contralesional hemispace. Patients with sensory neglect fail to perceive stimuli on that hemispace. Representational neglect typically has patients omit the contralesional hemispace when imaging or recalling scenes from memory. Second, USN can also be heterogeneous in the way it affects different levels of perception/representation. Patients’ personal space (patient’s own body), peripersonal space (within arm’s reach) and extrapersonal space (beyond reach) may not all exhibit symptoms with the same degree of affection. A subdivision along the spatial reference frame, i.e., egocentric (body-centered) or allocentric (object-centered), may also exist in some cases. These extremely various clinical subtypes of USN may exhibit different recovery trajectories and levels of severity [32], further complicating diagnosis and rehabilitation strategies. This clinical heterogeneity is closely linked to the neuroanatomical substrates and hemispheric asymmetries governing spatial attention and awareness.

#### 2.1.2. Hemispheric Asymmetries and Neuroanatomical Mechanisms of Attention

The variability of USN subtypes has been linked to distinct lesion patterns, most frequently involving frontal and parietal regions, particularly the right temporoparietal junction, which plays a key role in attentional control. In contrast, lesions in primary visual areas (occipital cortex) are rarely implicated [9,31]. While USN can result from damage to either cerebral hemisphere, left-sided USN following right hemisphere lesions (left USN) is typically more severe and persistent [33]. Right and left USN may appear with similar frequency during the acute phase but left USN tend to predominate in chronic stages [9,33,34]. This asymmetry has been attributed to the dominant role of the right hemisphere in mediating spatial attention and awareness [6,35].

This lateralization is supported by neuroanatomical models of attentional control involving two interacting cortical networks: the dorsal attention network (DAN), responsible for goal-directed spatial attention, and the ventral attention network (VAN), which mediates stimulus-driven reorienting [9]. While the DAN involves bilateral parietal and frontal cerebral structures, the VAN is right-lateralized, anchored in the right temporoparietal junction and frontal cortex. Damage to these regions can disrupt the integration between VAN and DAN, leading to profound attentional deficits [36]. Specifically, damage to the VAN, following right hemisphere stroke, is particularly disruptive due to the lack of compensatory counterpart in the left hemisphere. In contrast, the DAN’s bilateral architecture allows for partial functional compensation following unilateral damage, making VAN disruption a more critical factor in persistent neglect [35]. Consequently, right hemisphere lesions can critically impair attentional reorienting, leading to more severe neglect.

This model of USN is supported by functional imaging studies showing that USN severity correlates with decreased connectivity within these networks [36]. Specifically, the reorganization of attentional networks was positively associated with recovery mechanisms occurring during rehabilitation. These include reperfusion of critical cortical areas and compensatory recruitment of homologous regions in the contralesional hemisphere [37]. Notably, recovery would be more likely when the VAN remains structurally intact during the acute phase, whereas chronic neglect would be associated with persistent network disruption. These results underscore the importance of considering network-level dysfunction when designing rehabilitation strategies. For instance, effective interventions could aim to stimulate both stimulus-driven and goal-directed attentional systems to restore the balance between VAN and DAN, which is crucial for functional recovery. Additionally, recording and monitoring the neural correlates of these attentional processes could guide the personalization of interventions. This would help ensure that rehabilitation targets the underlying mechanisms rather than only behavioral symptoms. These recovery patterns are underpinned by neuroplasticity, defined as the brain’s capacity to reorganize its structure and function in response to experience, injury, or training [38]. In the context of USN, neuroplastic changes may involve the recruitment of contralesional networks, strengthening of residual pathways, or functional rebalancing between hemispheres [37,39,40].

Although substantial progress has been made in elucidating the neural basis of USN (e.g., the role of attentional networks disruption), the current understanding remains incomplete, especially regarding the temporal dynamics of the recovery mechanisms and their link and translation into functional recovery [41]. Research progress on these adaptive processes remains essential for improving the design of future targeted rehabilitation strategies.

### 2.2. Current Rehabilitation Strategies and Limitations

Although some patients experience spontaneous recovery from USN, symptoms persist beyond the acute phase in approximately one third of cases [42]. Given the severe functional consequences of USN and the heterogeneity of its manifestations, a variety of rehabilitation approaches have been developed [10].

Prism adaptation involves patients wearing optical prisms that laterally shift the visual field toward the contralesional side during visuomotor tasks. This manipulation provokes automatic changes in spatial representation [43]. Prism adaptation has been shown to reduce USN by inducing temporary modifications in sensorimotor systems. These changes extend to the cognitive level, leading to improvements in spatial attention and ameliorating both visuomotor responses and higher-level spatial representation [43,44]. Visual scanning training is another key intervention that teaches patients to systematically scan their environment, encouraging them to actively explore the neglected hemispace. This approach has demonstrated significant improvements in visual perceptual processing in USN patients [44,45]. Numerous other promising interventions have been proposed for USN recovery, including neuromodulation techniques (e.g., transcutaneous electrical nerve stimulation to stimulate somatosensory input) [46], motor-based therapies (e.g., contralesional limb activation) [47], and cognitive strategies (e.g., mental practice of contralesional limb movements) [48] (for review see [49]).

However, these approaches face major limitations due to the heterogeneity of USN. Patients with different subtypes or severities often respond differently to the same intervention [44] and evidence for long-term efficacy remains modest [41]. Interestingly, current clinical assessments often fail to fully capture the complexity of USN too, making it difficult to tailor interventions effectively and to propose clinically relevant rehabilitation protocols [50].

Furthermore, conventional interventions employed in clinical settings frequently lack ecological validity, with limited transfer of trained skills to real-world activities, and may fail to address specific subtypes of neglect [41,51]. This variability has underscored the need for more specific, individualized, and multimodal rehabilitation protocols [52].

Another prominent limitation of traditional rehabilitation approaches for USN, despite their widespread use, lies in the fact that they are often delivered through direct interactions with a therapist and lack objective progress quantification [49,53]. The lack of standardization and automation limits their reproducibility, adaptability, and precision in monitoring patient progress.

These limitations have driven the exploration of innovative computerized approaches aimed at overcoming the challenges posed by traditional therapies. Such methods offer an adaptive, engaging, and scalable platform for rehabilitation. Specifically, virtual reality (VR)-based interventions have emerged as a particularly promising avenue for enriching the possibilities of rehabilitation.

## 3. VR for USN Rehabilitation

### 3.1. What Is VR?

VR is broadly defined as computer-generated environments that simulate real or imagined settings, allowing users to interact with them virtually in real time. A key concept in VR is immersion, which refers to the level of sensory fidelity provided by the system. This often correlates with the user’s sense of presence (the psychological state of “being there” in the virtual environment), which contributes to user engagement [54,55]. Among the diverse VR configurations, fully immersive systems are particularly relevant in neurorehabilitation for their ability to engage multisensory modalities. These systems typically rely on head-mounted displays to create three-dimensional environments in which users are immersed across visual, auditory, and vestibular, proprioceptive sensory dimensions. Given their capacity to deliver interactive and highly engaging experiences, this review primarily focuses on immersive VR systems.

### 3.2. Interest of VR for Neurorehabilitation

Immersive VR offers a set of core features that make it particularly valuable for neurorehabilitation. Unlike conventional approaches, VR enables the simulation of ecologically valid scenarios within safe and controlled environments, allowing patients to engage in functional tasks without risks, and enhancing skill transfer to daily life [54,56]. Its capacity for precise and dynamic control over spatial, temporal, and sensory variables enables tailored restorative interventions that challenge specific cognitive functions and promote neural reorganization. The easily reconfigurable nature of virtual environments is particularly efficient at promoting targeted, repetitive, yet contextually varied, training for the patient.

In addition to ecological validity and safety, immersive VR systems allow for the recording of complementary behavioral and physiological metrics (e.g., eye and head movements, spatial navigation patterns and reaction time) contingent to task execution. In comparison to conventional setups, these complementary recordings provide valuable objective insights into motor and cognitive processes throughout therapy and support personalized adjustments.

Another key asset of immersive VR is the induction of a strong sense of presence, promoting engagement and adherence, which are crucial factors for positive rehabilitation outcomes. VR can also elicit illusory ownership and agency over a virtual body, allowing users to perceive the avatar’s movements as their own [57]. Such an embodiment effect has been shown to enhance motor recovery and cognitive engagement in post-stroke and neuropsychological rehabilitation contexts [58].

Importantly, VR also supports motivational engagement through the gamification of interventions. Gamified features such as reward systems and progressive challenges can transform repetitive rehabilitation tasks into engaging enjoyable experiences [59,60]. This is particularly important for long-term adherence in the context of rehabilitation protocols. Repeated exposure to virtual environments that simulate everyday contexts can reinforce compensatory strategies and facilitate the recruitment of contralesional brain regions. This is particularly effective when embedded in gamified scenarios [54,58].

These features collectively contribute to VR’s capacity to stimulate neuroplasticity and support functional recovery [54,55]. However, its increased efficacy compared to conventional cognitive neurorehabilitation methods has yet to be demonstrated [61].

Overall, these assets position VR as a promising tool for overcoming key limitations of conventional intervention in USN, including the lack of automation, limited ecological validity and insufficient objective progress tracking.

### 3.3. VR-Based Interventions for USN Rehabilitation

As mentioned in Section 3.2, traditional USN rehabilitation strategies, while beneficial, often lack ecological validity and fail to transfer to real-world situations [41,62]. In this context, VR has emerged as a promising tool to augment conventional USN interventions and address their limitations, with encouraging clinical outcomes [55,63,64].

As VR gained traction in neurorehabilitation, researchers have investigated whether VR-based adaptations of conventional USN rehabilitation strategies could match or even enhance their established therapeutic effects. Prism adaptation, for instance, has been successfully implemented in immersive VR setups. Studies conducted on neurotypical participants have shown that virtual prism adaptation could replicate the sensorimotor aftereffects of the conventional intervention [53,65] and induce changes in cortical connectivity associated with attentional networks [66], supporting its potential for clinical application. While most research on virtual prism adaptation has been conducted on neurotypical participants [67], there is recent evidence of effective sensorimotor bias induction towards the contralesional hemispace in patients with USN [68,69]. Similarly, clinical studies have shown that VR-based visual scanning training can enhance attentional engagement and spatial awareness in patients with USN [70]. According to therapist evaluations, gamified VR scanning tools hold strong potential to tailor rehabilitation to individual needs, enhance motivation, and sustain exploratory engagement [71].

In recent years, several immersive VR platforms have been developed to support USN rehabilitation, such as RehAtt [22], KF-SRT [72], or HEMIRehApp [73]. Unlike isolated VR tasks, these platforms represent scalable systems designed to deliver a range of interventions, within a unified technological framework. Preliminary results suggest that these modular systems may outperform conventional rehabilitation in terms of patient engagement, adherence, and USN symptoms reduction [22,69,72,73], while also facilitating therapist-guided monitoring [72,73]. Their design reflects the broader effort in neurorehabilitation to bridge experimental innovation with clinical implementation, supporting the transition toward more flexible and patient-centered therapeutic strategies.

While these developments are promising, most studies remain preliminary and share methodological constraints (e.g., healthy participants, small sample sizes, lack of control conditions), which are discussed in detail in the next section (Section 3.4).

The primary strength of VR in the rehabilitation of USN lies in its ability to address the condition’s heterogeneity. As mentioned in Section 2.1.1, USN manifests across multiple sensory modalities, spatial domains, and reference frames. VR environments offer the flexibility to simulate and manipulate these dimensions. For instance, VR-based methods for evaluating USN severity engaging several spatial regions and attentional processes have shown better diagnostic sensitivity than standard two-dimensional assessment methods [74,75,76,77]. Similarly, VR-based rehabilitation studies that specifically manipulate spatial domains have shown promising improvements in functional outcomes such as exploratory behaviors, attentional engagement and spatial awareness [76,78].

Moreover, VR search tasks integrating multimodal stimulation (e.g., visual, auditory, or proprioceptive cues), have been demonstrated to further enhance spatial awareness and encourage exploration of the neglected hemispace [22,60,70,79,80]. The intentional use of gamified elements designed to bias attention towards the neglected (contralesional) hemispace—such as asymmetric reward systems or spatially biased cueing—has shown potential to modify exploration pattern [81] and improve sustained attentional efforts in patients with USN [60,82].

Finally, immersive and adaptive VR environments offer unique opportunities to repeatedly engage patients in spatial exploration and attentional tasks, which are theorized to stimulate neural reorganization and functional recovery [55,83]. However, most studies investigating VR-based USN rehabilitation primarily focus on behavioral and clinical outcomes, with only a few directly measuring changes in brain connectivity or activation patterns to explain the underlying mechanisms of rehabilitation. Recent work demonstrated that improved exploratory behaviors following VR practice (e.g., increased scanning toward the neglected hemispace and more balanced visual search patterns) were associated with increased interhemispheric neural activity within the dorsal attention network (DAN) in chronic patients [84]. This evidence confirms the promising potential of VR-based protocols to reshape disrupted attentional networks at the origin of USN symptoms.

### 3.4. Limitations

Despite its promising potential, the use of VR in the rehabilitation of USN faces several limitations that must be addressed to ensure broader clinical adoption and efficacy.

One major challenge is the lack of standardized protocols across studies. For a structured summary of the main VR-based USN interventions referenced in Section 3.3 and their methodological characteristics, see Appendix A.1 (Table A1). The heterogeneity of VR setups, ranging from non-immersive desktop systems to fully immersive head-mounted displays, complicates the comparison of outcomes and the drawing of generalized conclusions. Moreover, sample sizes in existing studies are often small, long-term follow-up data are scarce, and there is a lack of control to compare VR efficacy to similar non-VR interventions [64].

Cybersickness and cognitive fatigue also pose usability challenges, especially for older patients or those with severe impairments (for a systematic review, see [85]). Although recent studies report good acceptance of VR interventions [73,86,87], further work is required to optimize user experience and minimize adverse effects. Key strategies include thoughtful design of virtual environments and interaction modalities.

Another concern could be the limited integration of neurophysiological monitoring within VR studies. While VR offers rich behavioral data, few studies incorporate neuroimaging or electrophysiological measures to track and validate the neural correlates of observed improvements (e.g., brain connectivity before vs. after VR intervention) [55,63]. This gap may hinder our understanding of how VR interventions modulate brain activity and which neural mechanisms are most responsive to virtual training.

Future research could benefit from conducting large-scale randomized controlled trials, integrating evaluation of transfer to daily life activities, developing adaptive VR environments for sensitive populations, and exploring applications beyond the laboratory setup.

These directions align with a broader shift in neurorehabilitation toward brain-centered, top-down strategies that emphasize the modulation of neural mechanisms underlying recovery. In this context, BCIs represent a compelling complement to VR, offering a unique opportunity to monitor and modulate neural activity in real time.

## 4. BCI for USN Rehabilitation

### 4.1. What Is a BCI?

BCIs enable direct control over the user’s external environment through their brain activity, offering unprecedented opportunities for rehabilitation and cognitive enhancement. Primarily designed to replace, restore, supplement or improve cognitive and motor functions, BCI technology has rapidly gained traction in the clinical field [13,88].

These systems capture, process, and translate the user’s neural signals into real-time actionable outputs, such as cursor movement or item selection. This output is then displayed through the interface to offer the user direct feedback, creating a closed-loop system between the brain and the device. By enabling interaction through neural signals alone, BCIs offer a unique pathway to re-engage disrupted brain networks. This approach can support recovery even in cases of minimal physical responsiveness, which is particularly valuable in the context of post-stroke rehabilitation [89,90]. Such direct engagement positions BCI technology as a powerful tool for promoting neuroplasticity and functional recovery.

Recent technological improvements have facilitated the evolution of BCIs from simple communication tools into more versatile, portable and sophisticated platforms capable of supporting cognitive enhancement and rehabilitation. This shift builds upon broader initiatives such as the mobile brain/body imaging (MoBI) approach, which advocates for the use of portable brain imaging systems in ecologically valid environments to study brain dynamics during naturalistic behavior. Often combined with other biometric measures, such approaches advocate to expand the applicability of neurotechnologies beyond controlled research settings [91]. They have triggered both hardware and software innovations to improve signal acquisition, processing, and classification in these more challenging conditions, particularly through the integration of artificial intelligence (AI, machine learning algorithms). Thereby, these advancements have enabled more precise targeting of neural activity and enhanced user engagement, increasing the potential to trigger neuroplastic changes.

### 4.2. Neuroplasticity Promotion

BCIs bypass traditional motor pathways and instead rely on decoding neural signals to interact with the environment. This closed-loop interaction fosters cortical re-engagement and supports the reshaping of disrupted neural networks in brain-damaged patients.

As highlighted by Mane et al. [13], BCIs facilitate neuroplasticity through four key mechanisms: (1) neurofeedback training, which allows users to learn how to consciously modulate their brain activity with real-time feedback; (2) operant conditioning, where contingent feedback reinforces desired neural patterns; (3) repetitive engagement of targeted brain regions; (4) Hebbian learning, which strengthens synaptic connections through neural co-activation.

Although most clinical applications of BCIs have targeted motor recovery, these mechanisms are equally relevant to cognitive domains, where growing research supports their potential for modulating cognitive functions. Specifically, BCIs have demonstrated efficacy in enhancing awareness and regulating activity within distributed attentional networks, including the parietal and frontal cortices, which are often disrupted in USN [83,88]. By engaging the brain in feedback-driven tasks continuously, BCIs create a learning environment supporting reorganization across cortical and subcortical structures, adaptive to each individual profile. Indeed, systems that dynamically adjust feedback or task parameters based on ongoing brain activity have been suggested to enhance the specificity and efficacy of cognitive rehabilitation [39,92].

### 4.3. Choosing the Right Neuroimaging Technique for a BCI

BCIs intrinsically rely on a neuroimaging technique to capture brain signals of the users. The available options can be categorized along several key dimensions, such as temporal and spatial resolution, invasiveness, portability, signal quality, and cost, that ultimately influence their clinical and practical applicability [93].

Contemporary research in BCI, including applications to neurorehabilitation, increasingly favors accessible, user-friendly systems that can be easily integrated in clinical settings or deployed outside the laboratory. Non-invasive techniques are generally preferred for these purposes. Advanced imaging techniques, including magnetoencephalography and functional magnetic resonance imaging offer rich temporal or spatial information but remain costly and non-portable, restricting their application to specialized research environments [94,95].

Despite its lower spatial resolution and signal quality, electroencephalography (EEG) combines affordability, portability and ease of use. As a portable, non-invasive technique that records electrical brain activity from the scalp, EEG currently offers the most practical neuroimaging solution for widespread clinical and research use, with numerous promising applications, including neurorehabilitation [93,96,97]. Consequently, we will focus on practical EEG-based BCIs for the remainder of the review.

### 4.4. Relevant EEG Biomarkers in USN for BCI Applications

Along with technological progress, EEG has become a valuable tool for investigating biomarkers of cognitive disorders, providing a non-invasive window into disrupted cortical dynamics. In patients with USN, EEG studies have revealed specific neurological markers and patterns of altered brain activity that correlate with behavioral symptoms and recovery trajectories [40,98].

Oscillatory activity analysis, which examines patterns of neural activity across frequency bands, provides valuable insights into these disruptions. Notably, abnormalities in alpha rhythm (8–12 Hz) hold strong diagnostic and prognostic value in USN [98]. During attentional tasks, patients exhibit a pathological increase in alpha power over the damaged hemisphere (parietal and occipital cortices). This abnormal activity reflects impaired attentional modulation and failure to inhibit ipsilesional hemisphere activity (see Section 2.1.2 on DAN/VAN asymmetries), leading to reduced responsiveness to contralesional stimuli [99]. Consistently, during resting-state EEG recording, patients with USN exhibit decreased alpha power in the damaged hemisphere, alongside increased slow-wave activity in posterior regions. This altered activity associated with disrupted network connectivity and attentional disengagement [100]. Together, these contrasting alpha patterns can serve as sensitive clinical biomarkers of attentional dysfunction and network-level disruption in USN. Beyond spectral power, quantitative EEG metrics such as the delta/alpha ratio and the brain symmetry index have also been proposed as objective biomarkers of USN. For instance, elevated delta/alpha ratio and increased asymmetry in parieto-occipital alpha activity seem to correlate with neglect severity and poorer performance on clinical tasks [100,101]. Collectively, frequency-band EEG measures offer non-invasive, functionally relevant markers of attentional bias and spatial awareness deficits in USN, with potential for diagnosis and rehabilitation monitoring.

Visual evoked potentials (VEPs), a subtype of event-related potentials (ERPs) specifically elicited by visual stimuli, have emerged as key EEG biomarkers for objectively detecting USN. VEPs offer high temporal resolution for tracking early visual and attentional processing, making them particularly suited for studying the neural mechanisms underlying USN [102]. VEPs recorded from USN patients often show increased latencies, particularly for contralesional stimuli [102,103,104]. These delays are most pronounced in late VEP components (e.g., N1p and P2) which are associated with top-down feedback and reactivation of dorsal parietal and occipital visual areas [102]. Absence or attenuation of these components for contralesional stimuli reflects the impaired attentional reorienting mechanisms observed in USN patients. In healthy individuals, reduced VEP amplitudes similarly correlates with decreased voluntary attention [105,106], further supporting the interpretation that amplitude reductions in USN indicate attentional dysfunction rather than sensory extinction. This reinforces the value of VEP characteristics as objective EEG markers of attentional disorders like USN. Moreover, VEP hemispheric asymmetries tend shown to normalize with rehabilitation, indicating their potential as indicators of USN recovery [107].

Beyond their clinical value in USN case, these EEG markers are highly relevant for BCI design. Many established BCI paradigms leverage these neural signatures (i.e., oscillatory dynamics and evoked potentials) to enable real-time monitoring, adaptive feedback, and user interaction in rehabilitative contexts [16,96]. This underscores their potential for rehabilitation-oriented BCI applications in USN.

### 4.5. BCI for the Rehabilitation of USN

Although several neural markers of USN have been identified, and a variety of EEG-based BCI paradigms are available [96], there is limited research on rehabilitation interventions integrating BCI technology for USN.

Tonin et al. [39] explored the feasibility of a BCI based on covert visuospatial attention (CVSA) in three patients with chronic USN. The system enabled patients to interact with visual stimuli by covertly shifting attention (i.e., without eye movements) toward the neglected hemispace, with real-time feedback derived from alpha-band activity analysis. Two patients showed significant improvements in reaction time for contralesional targets. These behavioral improvements were accompanied by cortical changes, such as increased alpha power in the affected hemisphere, reduced interhemispheric asymmetry, and enhanced functional connectivity in right parietal and occipital regions associated with attention control. These findings suggest that CVSA-based BCI may modulate cortical dynamics associated with attention control and hemispheric balance, which are key mechanisms involved in USN. While preliminary, the study offers a compelling proof-of-concept for BCI-driven cognitive rehabilitation, though further controlled trials are needed to establish its efficacy and generalizability.

Ros et al. [108] and Saj et al. [109,110] investigated the feasibility of EEG-based neurofeedback to alleviate USN symptoms by modulating alpha-band neural activity. These studies relied on implicit learning rather than explicit task performance, guiding patients to self-regulate brain activity using their own cognitive strategies through real-time visual feedback. In Ros et al., five patients with left-sided USN underwent six sessions of neurofeedback (NF) aimed at downregulating alpha activity in the right posterior parietal cortex [108]. Patients successfully learned to modulate their neural signals, and improvements in neglect symptoms were associated with increased alpha rhythm flexibility. Saj et al. applied a single early NF session in acute-phase left-sided USN patients, showing partial restoration of alpha dynamics and behavioral improvements [109]. While preliminary, these findings suggest that EEG-based NF can engage top-down attentional mechanisms and is a promising BCI approach for promoting attentional rebalancing and neuroplasticity in neglect recovery, in a patient-driven manner.

BCI applications for USN rehabilitation show early promise but remain limited by small sample sizes, lack of control conditions, and short-term assessments. For a summary of the BCI interventions discussed and their methodological characteristics, see Appendix A.2 (Table A2). Both NF and CVSA BCI paradigms have demonstrated feasibility and potential for modulating attentional networks altered in USN. However, therapeutic impact remains uncertain due to modest performance, limited follow-up data, and absence of standardized outcome measures.

Nonetheless, these preliminary results align with the broader neuroplasticity framework, facilitated by BCI technology, which posits that experience-driven modulation of synaptic strength and connectivity can restore functional integrity across distributed brain systems [111]. They support the use of EEG-based BCI as a promising non-invasive tool to promote adaptive neural reorganization in patients with USN.

### 4.6. Current Limitations and Prospects

#### 4.6.1. Limitations for BCI Integration

BCI solutions for USN rehabilitation remain underexplored, despite the technology’s promising potential for functional and cognitive enhancement in neurorehabilitation. This limited uptake may be partly explained by general EEG-based BCI implementation challenges, especially for interventions in brain-damaged populations.

Possible barriers include BCI illiteracy, or inefficiency. This concept refers to users’ inability to achieve reliable control over a BCI under standard training conditions. Importantly, it also encompasses the inherent challenges faced by classification algorithms to adapt to individual neural variability, particularly in motor imagery (MI) paradigms, limiting their effectiveness [112].

Other limitations involve the cognitive workload and attentional demands associated with BCI use and training, which vary depending on the BCI paradigm and task design. For instance, MI-based BCIs often require sustained concentration and mental effort (see Section 4.6.2), which can be challenging for users, particularly those with neurological impairments. These demands may reduce engagement and increase mental fatigue, thereby limiting the effectiveness of the intervention, as fatigue and disengagement can impair reliable interaction with the system [113].

Additionally, some paradigms require complex setups, involving the placement of numerous electrodes to capture specific neural activity. This can be time-consuming, and uncomfortable, and eventually hinder usability in clinical settings [114]. The variability of lesions sites in post-stroke populations further complicates BCI effectiveness. Lesions affecting different cortical or subcortical regions can alter the neural signatures targeted by BCI algorithms. Classification methods that rely on widespread networks activity or precise sensorimotor markers are particularly vulnerable. Such extended lesions often lead to reduced classification accuracy and limit generalizability across patients [27]. This concern is especially relevant for USN, where patients exhibit highly heterogeneous brain lesions (see Section 2.1.2).

However, these limitations are being progressively addressed through technological advances in BCI and neuro-engineering. Improvements in design, signal processing, and machine learning alongside the integration of AI-driven models, have led to more intuitive systems. These systems offer reduced training requirements, enhanced accuracy, better generalization across users, and an improved overall user experience [115,116,117,118].

#### 4.6.2. Underexplored BCI Paradigms for the Rehabilitation of USN

Several underexplored BCI paradigms offer promising avenues for future development in the rehabilitation of USN. These approaches aim to leverage neuroplasticity, enhance attentional engagement, and provide adaptive feedback to support recovery.

The motor imagery (MI) paradigm involves mentally rehearsing movements to activate motor and attentional networks without physical execution. MI-based BCIs have demonstrated potential for improving functional outcomes and facilitating neuroplastic changes in both sensorimotor and attentional networks. Studies showed that MI-based BCI helped reduce interhemispheric imbalance and improve attention-related mechanisms in post-stroke patients [13,119,120]. Although MI-based BCIs primarily target motor recovery, their influence on broader cognitive processes suggests potential relevance for USN rehabilitation. In a related non-BCI study, [48] investigated the use of kinesthetic visuomotor imagery as an add-on therapy for USN patients. Patients mentally rehearsed movements involving their contralesional upper limb, resulting in improvements in representational and visuospatial neglect symptoms, as well as in awareness of the affected limb. While this study did not involve a BCI, its structure could be naturally integrated into a closed-loop BCI framework to provide real-time feedback and enhance engagement.

Event-related potential (ERP)-based BCIs, particularly those using visual evoked potentials (VEPs) and late ERP components such as P300 (an endogenous “oddball” response related to attention and context updating), offer another promising direction. These components are well-established markers of attentional engagement and awareness, making them highly relevant for USN (see Section 4.4 for details). VEP-based BCIs leverage early visual cortex responses elicited by patterned, flickering, or transient stimuli. They enable direct assessment of visuo-attentional processing and are sensitive to attentional biases, even in minimal EEG setups [105,106]. Notably, in this context, VEP paradigms are less affected by lesion site variability, as USN patients rarely present damage in occipital visual regions (see Section 2.1.2). They are also less sensitive to BCI illiteracy, may require short training phase, and offer high usability [121]. Current research in VEP-based BCI focuses on optimizing visual stimulations (e.g., comfortable high-frequency or textured flickers) and EEG signals classifiers to improve both user experience and system performance [116,122,123,124,125,126,127]. Recent studies have proposed VEP-based BCI systems for the detection, spatial mapping, and severity assessment of USN [118,128]. While the diagnosis-to-rehabilitation transition of these systems remains largely unexplored, it is conceptually feasible.

## 5. Technological Combinations in USN Rehabilitation

### 5.1. Rationale for VR-BCI Integration

There is growing interest in hybrid neurotechnological interventions that combine BCIs with VR (VR-BCI systems) for their potential to stimulate cognitive recovery [83]. Such integration appears promising for USN rehabilitation and may overcome limitations of traditional interventions by embedding neurofeedback into ecologically relevant contexts, thereby enhancing both therapeutic efficacy and user motivation.

A strong theoretical rationale supports the synergy of VR-BCI systems. BCIs provide real-time feedback on cerebral states and activity, while VR offers immersive adaptive environments that can be tailored to individual cognitive profiles and challenge the brain in meaningful ways. Together, they enable dynamic learning systems that reinforce beneficial neural patterns and prune inefficient ones (see Section 3.2 and Section 4.2 for detailed neuroplasticity mechanisms).

By enabling patients to engage in naturalistic—yet controlled—tasks while monitoring their neural activity, VR-BCI applications offer tremendous opportunities to neurorehabilitation research [129]. The design of immersive virtual environments being extremely versatile, many different scenarios can be proposed to the patients to target the stimulation of specific brain regions, depending on their neurological condition. With the addition of the adaptive BCI feedback loop, VR-BCI systems can personalize the intervention to the patient’s neural activity and effectively modulate brain function—even at the neurochemical level. This may lead to improvements in cognitive functions such as memory, perception, and executive control in numerous cognitive enhancement contexts [83].

Importantly, recent evidence suggests that such hybrid systems may outperform BCI-alone or VR-only systems. For example, Blanco-Mora et al. [130] showed that integrating VR into MI-based BCI tasks significantly improved classifier performance across various EEG configurations. In this study, participants had to imagine rowing movements with one arm while observing the corresponding arm movement from a first-person VR perspective, as feedback. Compared to a screen-based version of the same task, the immersive setup resulted in stronger rehabilitative efficiency in participants. A follow-up functional imaging study adapted this paradigm and further revealed that VR-enhanced MI elicited broader and stronger brain activation in participants, particularly in visual–attentional and sensorimotor regions [131]. Although most plastic changes observed in VR-BCI interventions have been documented in motor rehabilitation contexts, some studies also report positive effects on cognitive states, including attention and executive functions [27,119]. Collectively, this suggests that cognitive rehabilitation could benefit from the same mechanisms (e.g., enhanced engagement, multisensory feedback, and targeted neuroplasticity) that drive motor improvements.

Furthermore, integrating BCI with VR could help address limitations identified in previous neurorehabilitation approaches involving VR. For instance, Salatino et al. [55] emphasized the lack of studies exploring the neural correlates of improved neglect symptoms after VR intervention and the need for continuous monitoring of the engagement of disrupted networks during therapy. A hybrid VR-BCI could fulfill this need by providing closed-loop feedback that dynamically adjusts task difficulty and stimuli based on real-time EEG markers of attention and engagement. Alternatively, it could simply enable brain activity monitoring concurrently with the VR task. Overall, integrating VR into existing BCI systems or vice versa represents a logical and promising evolution in neuro-rehabilitation.

### 5.2. Future of USN Rehabilitation: An Immersive BCI?

#### 5.2.1. Increased VR and BCI-like Systems in USN Detection

USN is a complex neuropsychological condition that affects patients’ processing of contralesional stimuli, with severe motor and cognitive consequences. Conventional diagnosis and rehabilitation approaches have shown limitations in addressing the diverse subtypes and manifestations of this syndrome. These shortcomings have prompted the exploration of novel technologies, among which, VR—including augmented reality (AR)—and BCIs have emerged as promising tools. While there is accumulating evidence for the benefits of VR intervention in such context (see Section 3.3), BCIs and BCI-like systems interventions are still in early development. By “BCI-like system”, we refer to systems that adopt principles of BCI paradigms without enabling active interaction between brain activity and an external device. Instead, neural responses are analyzed offline, typically for monitoring or assessment rather than real-time control. For example, a visual interface designed to elicit visual ERPs combined with EEG recording could track attentional states offline without providing feedback to the user. Such a BCI-like system typically serves as prior work for future interactive BCI.

Recent studies have explored the combined use of immersive technologies and EEG-based BCIs to assess spatial attention and awareness deficit severity in post-stroke patients with USN. Systems like AREEN [118,132] have demonstrated the feasibility of using immersive BCI-like technology to detect and assess neglect severity, including mapping the neglected visual hemispace. This system relies on processing EEG responses to lateralized visual stimuli presented in AR to estimate USN severity. These diagnostic capabilities support the rationale for extending BCI applications into rehabilitation, particularly when integrated with immersive technologies like VR or AR. Supporting this exploratory research, Eudave and Vourvopoulos [128] recently proposed a multimodal VR-based system combining mobile EEG and eye-tracking to map spatial attention in ecologically valid environments. While their proof-of-concept study was conducted on healthy participants and simulated USN, it represents an important methodological innovation, demonstrating the discrimination of attentional states through behavioral and neural markers. The authors suggest that multimodal data could enable real-time adaptation of VR tasks based on neurological responses. This approach would facilitate more personalized rehabilitation strategies for post-stroke patients with USN.

**Figure 1:**
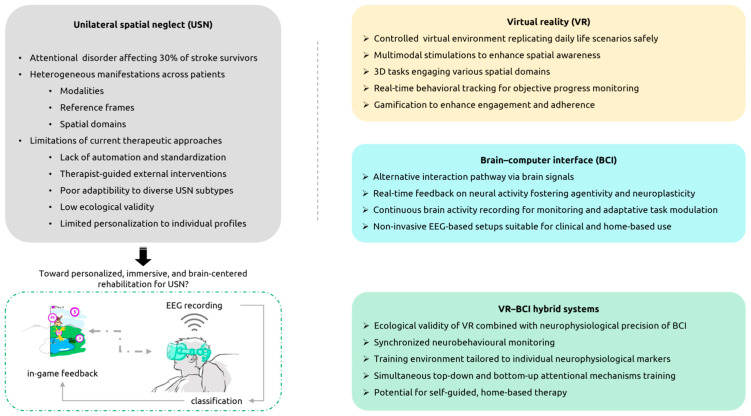
Overview of challenges in unilateral spatial neglect (USN) rehabilitation (e.g., symptom heterogeneity, limited ecological validity and limited adaptability of conventional rehabilitation) and emerging neurotechnological solutions that could address them. The figure illustrates the transition from conventional approaches to advanced strategies, including immersive virtual reality (VR), EEG-based brain–computer interfaces (BCIs), and integrated VR–BCI systems. These technologies could improve therapeutic efficacy by increasing patient engagement, enabling personalized interventions, promoting targeted neuroplasticity, and supporting autonomous use beyond clinical settings. Specifically, real-time adaptation of VR tasks based on neural data processing may further enhance engagement and rehabilitation outcomes.

Although emerging VR-BCI research focuses on USN assessment and monitoring (see Appendix A.3, Table A3), expanding previous pen-and-paper, and computer-based approaches, there is potential for rehabilitation interventions integrating both technologies (Figure 1). As mentioned in Section 4.6.2, researchers have already proposed BCI-only interventions to improve USN symptoms [39,108,109]. It is plausible that these paradigms could be adapted into immersive VR setups to amplify the therapeutic impact of real-time feedback and task engagement. Such integration could enhance neuroplasticity and support the restoration of altered brain activity patterns. This also applies to promising yet underexplored BCI paradigms in USN rehabilitation such as MI and VEP (see Section 4.6.2). This is further supported by the fact that MI, VEP, and NF paradigms have already been successfully embedded in virtual environments for motor rehabilitation or cognitive training tasks with enhanced performance [120,133,134,135,136].

#### 5.2.2. Actual Design Proposition—Integrated VR-BCI Approach for the Rehabilitation of USN

**Figure 2:**
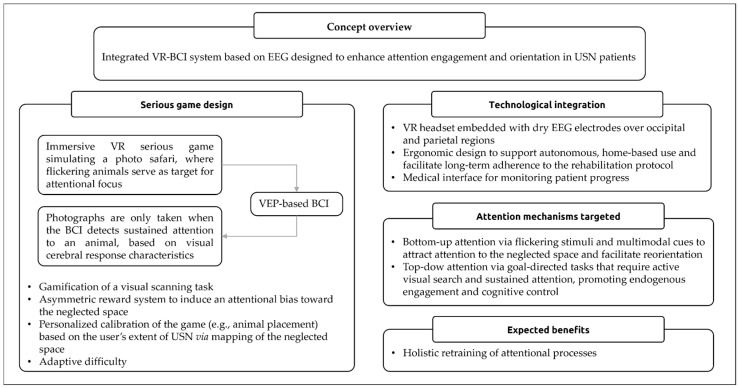
Summary of the proposed conceptual design for unilateral spatial neglect rehabilitation based on a virtual reality-embedded brain–computer interface [137]. Abbreviations: BCI, brain–computer interface; EEG, electroencephalography; USN, unilateral spatial neglect; VEP, visual evoked potential; VR, virtual reality.

We recently proposed a standalone therapeutic approach for chronic USN rehabilitation integrating a VEP-based BCI within an immersive VR environment, currently in development (see Figure 2) [137]. Its objective is to retrain disrupted attentional networks by engaging both bottom-up (stimulus-driven) and top-down (goal-directed) attention mechanisms. This is achieved through a gamified task combining visual scanning and attention reorientation, multimodal stimulations, and in-game real-time feedback on attentional engagement. VEP-based BCIs provide objective, precise measures of attentional engagement and offer direct assessment of visuo-attentional processing, making them particularly suitable for detecting the attentional biases observed in USN (see Section 4.4 and Section 4.6.2). VR environments complement this by offering immersive, ecologically valid contexts that can stimulate multiple cognitive processes and promote sustained engagement. Together, this approach exemplifies how VR and BCI technologies could be jointly leveraged to create engaging, scalable, and patient-centered rehabilitation tools suitable for specific cognitive disorders. While clinical validation is forthcoming, the system’s design aligns with current neuroscientific understanding of attention, reflects the growing trend toward user-friendly therapeutic device and offers a compelling direction for future development in neurorehabilitation.

Although this conceptual design promises to overcome several limitations of conventional rehabilitation, it remains a hypothetical solution requiring rigorous validation. In addition to general challenges of VR and BCI technologies (see Section 3.4 and Section 4.6), such an integrated approach faces specific implementation constraints, including hardware integration, signal quality, user comfort, and, most importantly, adaptability to diverse patient profiles. These aspects, along with broader usability and methodological issues, are discussed in detail in the next section.

#### 5.2.3. Limitations of VR-BCI Systems

While combining VR and BCI technologies holds great promise for neurorehabilitation, it also introduces specific technological constraints, beyond those already associated with each technology individually (see Section 3.4 and Section 4.6.1). These include challenges related to hardware integration, signal interference, cognitive demand, and overall usability.

Fully immersive virtual environments often induce more user movements (e.g., head and body movements), which are desirable for ecological validity but can generate motion artifacts in EEG recordings and reduce decoding accuracy. This issue is particularly problematic in simplified EEG systems with few electrodes, which are designed to reduce setup complexity. Although dry and wireless EEG solutions have facilitated sensor integration into VR headsets, they still face limitations in signal fidelity and user comfort. This is particularly important given the inherently poor signal quality of EEG.

Methodological limitations are another concern. Existing VR-BCI studies are frequently constrained by small sample sizes, lack of rigorous control designs, and protocol heterogeneity, which collectively hinder the generalizability and comparability of findings [83]. Most VR-BCI systems for USN rehabilitation are feasibility or proof-of-concept studies conducted on healthy participants (e.g., [128]) or conceptual frameworks, as the one presented here [137] (see Appendix A.3, Table A3 for a summary of the methodological characteristics of VR-BCI systems discussed in Section 5.2.1 and Section 5.2.3). While these exploratory efforts pave the way for promising rehabilitation protocols, they underscore the need for studies on actual patients with various profiles, large-scale randomized controlled trials and standardized protocols in this research field. Furthermore, long-term studies will be crucial to evaluate the clinical relevance of these interventions and assess the transfer of trained skills to daily life activities.

The immersive nature of VR can increase cognitive workload, particularly when users must simultaneously engage in mental tasks for BCI control while processing rich sensory stimuli. This multimodal demand may impair performance and reduce the reliability of BCI commands, thereby decreasing user engagement [138]. Designing interfaces that balance immersion with cognitive efficiency is essential to avoid overload and fatigue [139]. A critical factor in achieving this trade-off lies in the choice of BCI paradigms and the performance of classification algorithms (see Section 4.6.1). Paradigms requiring lower mental effort, minimal training and simple configurations are likely to foster greater engagement among users and improve overall BCI performance. However, in post-stroke rehabilitation contexts such as USN, lesion variability across patients introduces additional challenges. Damage to different cortical regions can affect the neural signatures targeted by BCI algorithms, reducing classification accuracy and limiting generalizability. This is particularly true for simplified systems relying on minimal EEG electrode configurations for improved usability. Although some BCI paradigms are less sensitive to BCI illiteracy and require lower effort (see Section 4.6.2), they could still be affected by patient diversity. For instance, visual stimulation-based paradigms are generally suitable for USN because occipital lesions are less common in this attentional deficit [9,31]. Yet, they can occur and make interaction with the device less effective for some patients. Choosing paradigms that minimize mental effort could improve engagement and performance, but this must be balanced with adaptability to individual profiles. Patient selection should be nuanced, with designs capable of accommodating multiple USN subtypes, diverse lesion characteristics, and varying individual cognitive profiles to optimize rehabilitation outcomes.

To mitigate these limitations, future designs could integrate multimodal physiological data to provide richer information for classification and faster detection of attentional states. For instance, combining eye-tracking with visually driven BCI paradigms based on EEG may accelerate signal interpretation and improve responsiveness (e.g., [128,140,141]). Advances in AI-driven algorithms also offer opportunities to improve adaptability and reduce training requirements (Section 4.6.1). These developments could help decrease sensitivity to patient profiles and support individually adaptive interventions, which will be crucial for clinical adoption and scalability. However, current research predominantly relies on highly controlled experimental settings that do not reflect real-world conditions. Evaluation of classification algorithms under ecologically valid, noisy conditions will be essential to ensure robust and reliable performance across diverse contexts, including clinical.

Another critical concern is the lack of systematic user experience evaluation and design validation in neurorehabilitation technologies. Future research would benefit from more structured user-centered methodologies (e.g., behavioral assessments) to ensure systems are not only effective but also usable, engaging, and acceptable to diverse patient and non-patient populations [126]. This includes evaluating emotional responses, perceived effort, subjective comfort and interaction quality, which are critical for long-term adherence and broader deployment of VR-BCI systems.

Finally, while VR and BCI technologies combination hold promise for USN rehabilitation, their clinical translation requires addressing practical considerations. These include clinician acceptance, patient autonomy, training burden and cost–benefit analysis. These technologies often require specialized hardware (e.g., VR headset with embedded EEG system) and software integration, presenting potential financial barriers. VR-BCI designs should consider long-term benefits, such as reduced therapist workload, improved patient autonomy, and decreased hospitalization time, that would help balance initial costs. For instance, integrating objective progress tracking, disorder monitoring (e.g., mapping neglect severity), or autonomous adaptive training while maintaining clinician intervention capabilities could enhance both efficiency and clinical relevance of such devices. Positive feedback from therapists on ease of use and integration into practice, together with compliance with regulatory pathways for digital therapeutics, will be essential to ensure that these systems can transition from experimental settings to routine clinical practice. Future research could benefit from explicitly incorporating these questions into design and evaluation frameworks, as they may be overlooked in existing studies.

## 6. Conclusions

USN remains one of the most challenging cognitive consequences of stroke, with profound implications for functional recovery and quality of life. As conventional rehabilitation approaches struggle to address the heterogeneity and persistence of USN symptoms, emerging neurotechnologies offer a compelling alternative. Current evidence suggests that VR can improve ecological validity, patient engagement, and attentional retraining. Similarly, EEG-based BCI interventions have demonstrated feasibility for modulating attentional networks and promoting neuroplasticity, although most findings stem from small-scale or proof-of-concept studies.

Despite these encouraging signals, several uncertainties persist. The comparative efficacy of VR and BCI interventions for USN versus conventional therapies remains unclear due to methodological limitations, such as small sample sizes, heterogeneous protocols, and limited long-term follow-up. The neural mechanisms underlying observed behavioral improvements after VR intervention are still underexplored. Moreover, promising BCI paradigms as well as hybrid VR–BCI systems, while conceptually compelling, have yet to be validated in clinical populations. Practical challenges, including signal quality, cognitive workload, and usability concerns further complicate clinical translation.

Future research in VR and BCI for USN rehabilitation should prioritize large-scale randomized controlled trials and standardized protocols with long-term outcome measures to confirm clinical benefits. Integrating multimodal monitoring to link behavioral gains with neurophysiological changes will help clarify underlying mechanisms and inform effective intervention design strategies. Another key priority will be the development of user-centered, adaptive designs that accommodate patient heterogeneity while minimizing cognitive load. Advances in artifact-resilient BCI pipelines, seamless hardware integration, and out-of-lab implementation will be essential to ensure scalability and accessibility in clinical practice.

In short, these neurotechnologies converge toward a shared objective: placing the brain at the center of the therapeutic process. By enabling immersive, adaptive, and feedback-driven interventions, these tools open new avenues for restoring attentional balance and promoting neuroplasticity. Although integration is still in its early stages, the growing body of evidence suggests that hybrid VR–BCI systems may represent a transformative step forward in USN rehabilitation and overall cognitive neurorehabilitation.

## 7. Methodological Statement

The present article did not adopt a systematic methodology when reviewing the existing literature on the addressed topics. This narrative review is representative of the authors’ accumulated knowledge of state-of-the-art research over the last years studying this field and aims to offer original perspectives to the reader. Nonetheless, for the purpose of this publication, they made sure to gather the most recent peer-reviewed articles indexed in PubMed and Scopus, employing “VR”, “BCI”, and “USN rehabilitation” (and their most common synonyms) as search terms. They acknowledge that this approach hinders reproducibility of the article selection process and the comprehensiveness of this work.

## Data Availability

No new data were created or analyzed in this study.

## Abbreviations

The following abbreviations are used in this manuscript:

AI	Artificial intelligence
AR	Augmented reality
BCI	Brain–computer interface
CVSA	Covert visuo-spatial attention
DAN	Dorsal attention network
EEG	Electroencephalography
ERP	Event-related potential
MI	Motor imagery
NF	Neurofeedback
USN	Unilateral spatial neglect
VAN	Ventral attention network
VEP	Visual evoked potential
VR	Virtual reality
VR-BCI	Virtual reality-based brain–computer interface

## Appendix A

### Appendix A.1

Table A1 summarizes key characteristics (study type, participants, intervention, assessment methods, main outcomes, follow-up, and presence of control group) of the main VR interventions for USN discussed in Section 3.3. Current literature exhibits important methodological constraints that limit the generalizability and clinical translation of findings, as highlighted in Table A1 and Section 3.4.

**Table A1:** Summary of the main VR interventions for USN discussed in Section 3.3. Abbreviations: USN, unilateral spatial neglect; fMRI, functional magnetic resonance imaging; RHS, right hemisphere stroke; CBS, Catherine Bergego scale (functional assessment of USN in daily activities); SCT, star cancellation test (pen-and-paper test for USN); BTT, baking tray task/test; LB, line bisection (pen-and-paper test for USN); EXT, extinction test; PA, prism adaptation; CAVE, cave automatic virtual environment (immersive VR system using projection walls); DAN, dorsal attention network; CLOX, clock drawing test; ADL, activities of daily living; NA, not applicable.

References	Study Type	Participants	Intervention	Assessment Methods	Main Outcomes	Follow-Up	Control Group
[22]	Multiple-baseline single-arm intervention	15 USN patients after RHS	VR visual scanning task with multisensory stimulation and visuomotor activation of the contralesional hand. 3 sessions/week (1 h each) for 5 weeks.	VR neglect test battery (SCT, BTT, LB, EXT, Posner cueing task); CBS; 3 baseline sessions, post-interventions and CBS at 6-month follow-up.	Significant improvements on all tests; sustained effect at 6-month follow-up (CBS).	Yes (6-month)	No (single-arm multiple-baseline design)
[53]	Proof-of-concept/Methodological validation	10 healthy participants	Immersive VR PA task; comparison of virtual and real prisms across three test modalities (real, mixed, virtual) with pointing tasks. 3 sessions per participant (one per modality).	Deviation angle in pointing tasks.	Virtual prisms induced aftereffects comparable to real prisms (leftward deviation); no retention effect at 2 h; VR system validated as a potential rehabilitation tool.	No (retention tested at 2 h post-exposure)	No (within subject comparison)
[56]	Pilot/case–control study	19 USN patients after RHS divided into two groups: VR-group *n* = 11, Control group *n* = 8	VR group: non-immersive virtual street-crossing training; Control group: standard computer-based visual scanning task. 12 sessions over 4 weeks.	Pen-and-paper tests, ADL checklist; performance on a virtual street-crossing task; real-world street-crossing performance. Pre-/post-intervention.	VR and Control groups improved equally on standard USN measures. VR group outperformed controls on the VR test and some real-world street-crossing.	No	Yes
[60]	Feasibility study	15 healthy controls (Phase 1); 7 stroke patients with/without USN (Phase 2–3)	Immersive VR game; gamified visual discrimination tasks in naturalistic scenes with patient-tailored multisensory cues. Phase 1–2: stroke patients and healthy controls, single session; Phase 3: USN patients, 6 sessions over 6 days.	Simulator sickness questionnaire; user experience ratings; behavioral inattention test, pen-and-paper/computerized cancellation task, figure copy; in-game performance metrics.	VR game feasible and well tolerated; USN symptoms accurately detected in VR tasks; multisensory cues improved contralesional target detection in USN patients.	No	No (within-subject comparisons; placebo game in some Phase)
[65]	Experimental controlled study	30 healthy participants divided into 3 groups (10 participants each): control, little interaction group, high interaction group.	Immersive VR PA pointing task; participants performed 0 (control), 5 (little interaction group) or 35 (high interaction group) pointing movements in adaptation phase depending on their group. Single session.	Deviation angle in pointing tasks (baseline, adaptation, readaptation phases); magnitude and persistence of aftereffect measured post-adaptation.	Results consistent with conventional PA studies; dose-dependent adaptation aftereffect in magnitude and persistence from 5 pointing movements during adaptation phase	No	Yes
[66]	Sham-controlled experimental study	45 healthy participants divided into 3 groups: rightward PA (*n* = 16), leftward PA (*n* = 14), sham (*n* = 14)	Immersive VR PA; gamified catching tasks with visuomotor rotation (rightward, leftward, or sham). Single session.	Behavioral aftereffects measures; fMRI connectivity pre/post adaptation (resting-state and naturalistic video viewing).	VR PA induced sensorimotor rightward/leftward; upregulation of parieto-occipital activity during naturalistic viewing after rightward PA; neural changes correlated with behavioral changes.	No (retention tested at 40 min post-adaptation)	Yes
[68]	Double-blind, within-subject study	15 patients with left USN after RHS	Immersive VR PA; pointing task with gradual rightward shift (0°, 15°, 30°) of controller image. Each patient completed one session per condition in randomized order.	Pointing deviation angle; LB, target cancellation pre/post adaptation; awareness questionnaire.	Dose-dependent adaptation effects in pointing deviation (greater leftward after-effect at 30°); no significant transfer to USN measures.	No	No (within-subject design: 0° deviation as control)
[70]	Randomized controlled trial	11 USN patients after RHS divided into 2 groups: training with VR visual exploration therapy then waiting without this training (TW, *n* = 5) or vice versa (WT, *n* = 6)	VR visual exploration task; patients had to detect targets among distractors, arranged in three-dimensional spherical spaces around them. Waiting: 4 weeks; training: 5 session/day over 4 weeks; similar conventional rehabilitation throughout the intervention period.	Clinical USN measurements (LB, SCT, CBS); VR-based metrics (field of perception and field of regard: response time, success rate, head movement).	Significant improvements on clinical tests performance; enhanced detection and response speed, particularly for affected hemispace.	No	No (waiting period)
[69]	Feasibility/case-series study	3 left-sided USN patients after stroke	Immersive VR reaching task with gradual visuomotor misalignment (prism adaptation inspired mechanisms). 5 sessions within 10 days.	BIT at 3 baselines; immediately post-intervention, and 2-week follow-up; user experience-related questionnaires.	Immediate improvement in test accuracy for all participants; sustained improvement at 2 weeks for 2 participants; good user experience.	Yes (2-week follow-up assessment)	No (multi-baseline design)
[72]	Usability study	10 USN patients after stroke, 5 clinicians	Development and iterative testing of immersive VR game-like tasks targeting neglected space (e.g., arm-reaching task with virtual visuomotor misalignment, head-turning and combined head/arm movements).	User experience-related questionnaires and interviews.	Patients reported good usability, comfort, and preference for VR over conventional therapy; no cybersickness; clinicians satisfied with interface. No USN outcome data.	NA	NA
[73]	Placebo-controlled longitudinal study	6 USN patients after RHS (2 completed full protocol)	Immersive VR game with multisensory stimulation; gamified visual discrimination tasks in naturalistic scenes; active condition with contralesional bias in target locations vs. placebo with central targets. 10 daily 1 h sessions per condition.	Posner cueing task; cancellation tests, LB, CBS, sustained attention task; in-game metrics; user experience and cybersickness metrics. 1-week follow-up.	Active VR training condition improved detection of left-sided targets within VR; no consistent transfer to non-VR tasks; minimal cybersickness.	Yes (1-week follow-up assessment)	Yes
[74]	Feasibility/case–control study	27 patients with RHS divided into 2 groups: USN^+^ (with USN, *n* = 12), USN^−^ (without USN, *n* = 15); 9 healthy controls	VR visual search and detection of items while navigating a virtual grocery store, either in a simple or cluttered scene. Single session.	Standard USN assessment (LB, SCT, apples test); detection and navigation task metrics (detection time, maximal mediolateral deviation from ideal trajectory, navigation time to target).	Worse performance in USN^+^ vs. USN^−^ on neglect measures, with longer detection times, larger mediolateral deviations, and slower navigation, particularly in cluttered scene; no difference between healthy controls and USN^−^; VR system detected deficits missed by conventional tests.	No	Yes
[75]	Pilot/case–control study	15 RHS patients divided into 2 groups: Neglect (with USN, *n* = 10) and No-Neglect (without USN, *n* = 5); 35 healthy controls	VR cancellation task of targets, among distractors, arranged in a three-dimensional space around the patient.	Pen-and-paper/VR cancellation tasks.	Neglect performed worse than No-Neglect and healthy controls on both tasks; similar performance in USN detection for VR and pen-and-paper tasks; good usability and acceptance of the VR task.	NA	Yes
[76]	Case report	1 acute USN patient after RHS	VR balloon detection in eight spatial quadrants; treatment involved repeated practice under tilted background condition.	Clinical USN assessment (CBS, conventional pen-and-paper tests); VR evaluation (reaction time to target appearance in left/right, upper/lower, proximal/distal spaces).	VR detected mild neglect missed by conventional tests; reaction time significantly slower for left and proximal space pre-treatment; improved awareness of left space post-treatment; upper space awareness improved.	No	No
[77]	Feasibility study	39 healthy participants	Immersive VR tasks assessing near and far space neglect in ecological environments.	VR metrics: exploration time, omissions, time-to-reach, reaction time, head/gaze/hand tracking; comparison with pen-and-paper tests.	VR tasks well tolerated; high usability and presence; very low omission rates; left/right asymmetries in exploration and reaction time.	No	No
[78]	Pilot/case-series study	10 USN patients after RHS	Immersive VR visual search task for far space, reaching task for near space; moving slit to promote leftward attention shift. Single session.	Line cancellation, SCT, letter cancellation, LB tests for near and far space pre/post session.	Significant improvement in far space and cancellation tasks; near space and LB unchanged.	No	No
[79]	Proof-of concept study	21 USN patients after RHS divided into 2 groups: Experiment I (*n* = 9), Experiment II (*n* = 12)	Auditory stimulation using preferred music with spatial cueing (moving right to left) vs. without cueing. Randomized single-blind cross-over design. Experiment I: 5 patients started with cueing condition; 1 session per condition on separate days. Experiment II: 7 patients started with cueing condition; 1 session per condition on separate days.	Standard USN assessment tests (letter cancellation, LB, CBS). Experiment I: letter cancellation pre/post auditory stimulation; Experiment II: free visual exploration with video-oculography before and 1 h/3 h post stimulation.	Auditory spatial cueing improved neglect vs. no cueing, with stronger and longer-lasting effects. Experiment I: cueing improved immediate letter cancellation score vs. no cue. Experiment II: cueing only led to leftward gaze shift at 3 h post-intervention; increased exploration area at both 1 h and 3 h post-intervention, mainly in contralesional space.	No	No
[80]	Pilot study	12 USN patients after RHS; 5 with somatosensory impairment, 7 without.	Immersive VR visual search task, patients had to search for birds located at 4 different angles around them; 4 conditions: no cue, auditory cue, tactile cue, combined audio-tactile cue. 1 session per condition, over 2 consecutive days.	Task performance metrics; early orientation; system usability scale; simulator sickness questionnaire	Cueing significantly improved performance vs. no cue, especially for contralesional targets; all cue types triggered better early orientation; no additive benefit of combined cues.	No	No (within-subject comparison)
[81]	Experimental study	41 healthy participants divided into 2 groups: high reward on left, high reward on right.	Immersive VR exploration using CAVE system; navigation with detection tasks followed by exploration task; asymmetric reward contingencies (high reward for targets on one side, low reward on the other). Single session.	Eye-tracking (gaze orientation); exploration trajectories; spatial choices at path trifurcation.	Reward learning in VR biased visual attention and eye movements toward high-rewarded side; left-side high rewards increased leftward gaze fixations and modest bias during exploration; no generalization to spatial choices.	No	No (group comparison, no sham condition)
[82]	Experimental study (within-subject design)	20 RHS patients divided into 2 groups: with USN (*n* = 10), without USN (*n* = 10)	Computerized visual search task with spatial cueing and reward learning: targets preceded by valid/invalid cues, distractors associated with high or low reward during training. Single session.	Reaction times to detect targets; effects of cue validity, target side, and distractor reward history; voxel-based lesion-symptom mapping.	Rewarded distractors strongly interfered with reorienting to left targets after invalid cues in USN group, especially when both were contralesional; effect correlated with USN severity; stronger reward bias linked to right angular gyrus and occipito-temporal lesions.	No	Yes
[84]		13 chronic USN patients after RHS	Immersive VR gamified scanning training (RehAtt) with multisensory stimulation and contralesional hand activation via haptic feedback. 5-week training.	Behavioral tests (Posner cueing task, SCT, EXT, BTT, CBS) pre/post training; resting-state fMRI connectivity pre/post training.	Improved neglect symptoms and increased interhemispheric functional connectivity within the DAN; DAN showed greater spatial remapping than other networks.	No	No
[86]	Proof-of-concept study	21 young healthy participants, 23 elderly healthy participants, 11 USN patients after RHS	Immersive VR visual search task in 360° naturalistic environment; moving targets tagged with handheld controller; adaptive difficulty scaling based on performance; patient version simplified (fewer distractors, slower targets). Single session.	Usability questionnaires; in-game behavioral metrics: mean search time, controller position, spatial exploration; USN test (cancellation task).	High usability across groups. USN patients showed typical rightward bias and prolonged search for left targets, correlated with severity; effective capture of neglect patterns.	No	No (healthy participants served as reference for usability, not as experimental controls)
[87]	Pilot study	13 brain injury patients including 3 with USN, 9 clinician controls	Immersive VR visual search and detection tasks; six levels with increasing complexity; normative modeling to detect visuospatial atypicality. Single session.	Standard USN assessment tests (CLOX, single letter cancellation, Albert’s test); VR metrics: accuracy, reaction time, headset/controller orientation; usability and motion sickness questionnaires.	VR task well tolerated in all groups; atypicality in VR metrics enabled identification of all patients with USN identified by standard tests as well as additional cases under-detected by conventional assessment.	No	Yes

While a wide range of strategies have been proposed, most studies are feasibility or proof-of-concept trials (preliminary designs of rehabilitative approaches), often involving fewer than 15 participants and sometimes may not include patients with USN. This restricts the ability to draw general conclusions and limits external validity. In addition, only a few studies include long-term follow-up, although monitoring for sustained effects and transfer to daily life is crucial in neurorehabilitation. The lack of rigorous control conditions across interventions (many studies adopted single-arm, case-series or within-subject designs without active control groups) make it difficult to isolate VR-specific effects from spontaneous recovery, conventional intervention or placebo influences. Moreover, variability in task design and outcome measures hinders cross-study comparisons and synthesis of evidence. Finally, although recent rehabilitation-focused studies have integrated usability questionnaires suggesting good acceptance, user-centered designs and systematic user experience evaluation remain underdeveloped.

### Appendix A.2

Table A2 summarizes key characteristics (study type, participants, intervention, assessment methods, main outcomes, follow-up, and presence of control group) of EEG-based BCI interventions for USN rehabilitation discussed in Section 4.5.

**Table A2:** Summary of BCI-based interventions for USN rehabilitation discussed in Section 4.5. Abbreviations: USN, unilateral spatial neglect; EEG, electroencephalography; fMRI, functional magnetic resonance imaging; RHS, right hemisphere stroke.

References	Study Type	Participants	Intervention	Assessment Methods	Main Outcomes	Follow-Up	Control Group
[39]	Feasibility study/case series	3 chronic USN patients after RHS	EEG-based BCI control via covert attention shifting toward neglected hemispace; patients maintained fixation and pressed a button at target selection by the BCI. 6 sessions over 2 weeks.	Reaction time and EEG markers (alpha power, connectivity).	Improved reaction time for 2 patients; increased ipsilesional alpha power; reduced inter-hemispheric imbalance in parieto-occipital regions; enhanced functional connectivity in affected hemisphere.	No	No
[108]	Feasibility study/case series	5 USN patients after RHS	EEG-based neurofeedback task targeting alpha rhythm downregulation at right posterior parietal cortex with visual feedback. 6 daily sessions over 1 week after 1-week baseline.	Alpha amplitude and variability; line bisection and bell cancellation tasks pre/post sessions.	Normalization of alpha variability post treatment; reduced omissions in neglected space; correlation between EEG changes and behavioral improvement.	No	No active control group (normative EEG comparison only)
[109]	Feasibility study/case series	4 acute USN patients after RHS	EEG-based neurofeedback task; patients trained to downregulate alpha rhythm at right posterior parietal cortex via game-like visual feedback. Single 30 min session.	Line bisection, bell cancellation, scene copying before, after, and 1 week post session.	Reduced alpha amplitude during neurofeedback task; improved neglect score immediately and at 1 week but no significant difference between baseline and 1 week.	Yes (1 week)	No active control group (normative EEG comparison only)
[110]	Perspective with preliminary case-series results	fMRI: 7 chronic USN patients; EEG: USN patients, 4 acute and small chronic group	fMRI: neurofeedback task to upregulate right occipital cortex activity (3 sessions over 3 weeks); EEG: neurofeedback task to downregulate alpha rhythm over right parietal cortex (acute: 1 session; chronic: 5 sessions over 1 week).	Line bisection, bell cancellation, scene copying; EEG/fMRI markers.	fMRI: reduction in USN severity after 3 sessions. EEG: acute group, immediate improvement but not sustained; chronic group, moderate improvement in cancellation tasks correlated with alpha variability normalization.	No	No active control group (normative EEG comparison only)

USN rehabilitation through BCI technology is still in its early stage; the body of evidence is very limited, with only a handful of feasibility studies available. These studies are characterized by important methodological limitations, including very small samples sizes, lack of active controls, variability in study design, and absence of long-term follow-up. Such constraints limit cross-study comparison and currently allow only the identification of promising results rather than firm conclusions regarding the effects of neurofeedback or attention-based paradigms effects. Although the small number of studies limits consistency across evaluation methods, most combine behavioral assessments of USN with neurophysiological markers. This parallel analysis suggests that BCI-driven interventions can promote normalization of neural network connectivity alongside improvements in USN behaviors, but confirmation in larger, controlled trials is needed.

### Appendix A.3

Table A3 summarizes key characteristics (study type, participants, intervention, assessment methods, main outcomes, follow-up, and presence of control group) of the main VR-BCI interventions for USN, as discussed in Section 5.2.1 and Section 5.2.2, including BCI-like systems.

**Table A3:** Summary of the main VR-BCI interventions for USN discussed in Section 5.2.1 and Section 5.2.2. Abbreviations: USN, unilateral spatial neglect; EEG, electroencephalography; VR, virtual reality; AR, augmented reality; VEP, visual evoked potential; ERP, event-related potential; NA, not applicable. Scope note: Table A3 includes VR-BCI (closed-loop/interactive) and VR-BCI-like (assessment/monitoring without user-visible real-time feedback) systems.

References	Study Type	Participants	Intervention	Assessment Methods	Main Outcomes	Follow-Up	Control Group
[118]	Methodological validation/Proof-of-concept (model development and validation)	21 stroke participants divided into 2 groups: with USN (*n* = 11), without USN (*n* = 10)	AR starry night task with concomitant EEG; evaluation of ESTNet deep learning model for mapping neglect severity across patient-specific field of view (post-session analysis).	Accuracy, sensitivity, specificity; saliency maps	High accuracy, sensitivity and specificity of the introduced model for detecting USN; potential as an effective tool for generalized USN assessment.	NA	
[128]	Proof-of-concept study	9 healthy participants	Visual detection task in VR; 2 conditions: control vs. simulated USN (Left Occlusion); integrated eye-tracking; concomitant EEG. Single session. Study exploring multimodal mapping of spatial attention.	Reaction time, eye-gaze, head and controller movements; EEG band power and ERP analysis.	Worse behavioral performance in Left Occlusion condition (longer reaction time and rightward gaze bias); differences in neural activity between the two conditions; the system could be used as a diagnostic virtual reality tool for USN.	No	No (within subject only)
[132]	Feasibility/case–control study	10 stroke participants divided into 2 groups: with USN (*n* = 5), without USN (*n* = 5).	AR starry night task with concomitant EEG (AREEN system); target detection among distractors. Single session. Study evaluating AREEN for USN detection capabilities (post hoc analysis).	Field of view mapping; EEG bandpower ratios; reaction time.	Accurate USN detection; accurate neglected target prediction in USN group based on EEG features.	NA	Yes
[137]	Conceptual design	NA	VR serious game integrating an EEG-based VEP BCI; visual scanning and detection task with gamified feedback; target capture via attention decoding. Adaptive interface/game to the patient profile.	Game performance metrics; neglected space mapping; EEG markers analysis.	Not yet tested.	NA	NA

To date, evidence of combined VR-BCI integration is at a very conceptual stage. Current evidence is limited to VR-BCI-like systems (e.g., use of BCI paradigm for monitoring only with post-session data analysis) for USN diagnostic or severity mapping feasibility in USN or healthy participants. Therapeutic VR-BCI designs for USN remain underexplored. The very small samples, the variability in outcome measures and the absence of comparison against conventional or VR-only/BCI-only assessment approaches represent major methodological limitations and constrain the generalizability of their promising diagnostic efficacy.
